# *Cynanchum wilfordii* Polysaccharides Suppress Dextran Sulfate Sodium-Induced Acute Colitis in Mice and the Production of Inflammatory Mediators from Macrophages

**DOI:** 10.1155/2017/3859856

**Published:** 2017-06-20

**Authors:** Chang-Won Cho, Sungeun Ahn, Tae-Gyu Lim, Hee-Do Hong, Young Kyoung Rhee, Deok-Chun Yang, Mi Jang

**Affiliations:** ^1^Traditional Food Research Center, Korea Food Research Institute, Seongnam, Gyeonggi 13539, Republic of Korea; ^2^Department of Oriental Medicinal Biotechnology, College of Life Sciences, Kyung Hee University, Yongin, Gyeonggi 17104, Republic of Korea

## Abstract

We recently reported the immune-enhancing effects of a high-molecular-weight fraction (HMF) of CW in macrophages and immunosuppressed mice, and this effect was attributed to a crude polysaccharide. As polysaccharides may also have anti-inflammatory functions, we investigated the anti-inflammatory effects and related molecular mechanisms of a crude polysaccharide (HMFO) obtained from HMF of CW in mice with dextran sulfate sodium- (DSS-) induced colitis and in lipopolysaccharide-induced RAW 264.7 macrophages. HMFO ameliorated the pathological characteristics of colitis and significantly reduced production of proinflammatory cytokines in the serum. Histological analysis indicated that HMFO improved the signs of histological damage such as abnormal crypts, crypt loss, and inflammatory cell infiltration induced by DSS. In addition, HMFO inhibited iNOS and COX-2 protein expression, as well as phosphorylated NF-*κ*B p65 levels in the colon tissue of mice with DSS-induced colitis. In macrophages, HMFO inhibited several cytokines and enzymes involved in inflammation such as prostaglandin E_2_, nitric oxide, tumor necrosis factor-*α*, interleukin-6, inducible nitric oxide synthase, and cyclooxygenase-2 by attenuating nuclear factor-*κ*B (NF-*κ*B) and mitogen-activated protein kinases. HMFO attenuated inflammation both in vitro and in vivo, primarily by inhibiting NF-*κ*B activation. Our findings indicate that HMFO is a promising remedy for treating inflammatory bowel diseases, such as colitis.

## 1. Introduction

Excessive environmental and industrial development has created hazardous conditions, such as viral diseases (e.g., Middle East respiratory syndrome (MERS)) [1] and particulate matter air pollution, which has been classified as carcinogenic to humans (IARC Group 1) [2]. Thus, the importance of building a strong immune system is becoming more important for good health, and the demand for health supplements that can help improve the immune system has been rapidly increasing. There is a close correlation between immune responses and inflammation. When immunity decreases, the body is easily exposed to infectious and noninfectious inflammatory diseases.

Ulcerative colitis (UC) is a type of inflammatory bowel disease (IBD). It is estimated that 1-2 million people in the United States suffer from IBD, approximately half of which have UC. The exact cause of UC was unknown, but it appears to be related to a combination of genetic and environmental factors [3]. The goal of conventional IBD therapies is mainly to downregulate aberrant immune responses and inflammatory cascades. Conventional drugs for IBD include corticosteroids and 5-aminosalicylic acid (5-ASA; mesalazine), but these drugs have undesirable side effects, especially steroid therapy, which can cause infertility and developmental disability [4, 5]. IBD patients present diverse symptoms throughout the course of the disease. Some symptoms are directly related to disease activity, while others may be a consequence of therapy. The disease imposes a substantial burden on patients and significantly impacts their functioning and health-related quality of life [6].

In spite of advances in modern medical science, traditional herbal medicine has continued to be widely used for health maintenance, disease prevention, and even disease treatment in China, Japan, and Korea for thousands of years. Previous studies have shown that 1 out of 2 IBD patients turns to complementary and alternative medicine [7–10]. *Cynanchum* is a genus that includes approximately 300 species that are distributed worldwide, including east Africa, the Mediterranean, the tropical zone of Europe, and the subtropical and temperate zones of Asia [11]. Many *Cynanchum* species have been used as traditional medicines in Korea to prevent and treat various diseases such as rheumatic arthritis and geriatric, vascular, and ischemia-induced diseases [12–14].

The roots of *Cynanchum wilfordii* Hemsley are referred to as *Cynanchi wilfordii* Radix in the Korean Pharmacopoeia [15]. *C. wilfordii* has received substantial attention as a traditional herbal medicine that can be used to treat various diseases. It has many potentially beneficial effects such as antitumor, antioxidant, and anti-inflammatory effects; guards against diabetes mellitus, gastric disorders, neuronal damage, and hypercholesterolemia; and causes vascular relaxation effects [16–21]. This plant is well-known to contain biologically active compounds, including gagaminine and its glycosides, wilfosides, and cynauricuosides, as well as sarcotine, penupogenin, and cynandione A [22].

Polysaccharides of plant origin have proven to be an important class of natural bioactive products in recent years [23], and previous studies have demonstrated that they possess antitumor [24], immunity-enhancing [25], anticoagulant, antithrombotic, antioxidant, and anti-inflammatory activities [26]. Previously, we showed that a high-molecular-weight fraction (HMF) of *C. wilfordii* exerted immune-enhancing effects on immunosuppression induced by cyclophosphamide and on macrophages, and this effect was attributed to the crude polysaccharide [25]. However, the anti-inflammatory effects of polysaccharides of *C. wilfordii* in a dextran sulfate sodium- (DSS-) induced mouse model of colitis and the related mechanism of action have not yet been reported. As polysaccharides may also have anti-inflammatory functions, we investigated the anti-inflammatory effects of polysaccharides from *C. wilfordii* on lipopolysaccharide- (LPS-) induced RAW 264.7 macrophages and on DSS-induced chronic colitis in mice.

## 2. Materials and Methods

### 2.1. A Crude Polysaccharide (HMFO) Preparation

HMFO was prepared as previously described [25]. Briefly, HMFO was obtained from a high-molecular-weight fraction of *C. wilfordii* that was processed through polyethersulfone ultrafiltration membranes with a molecular weight (M.W.) cutoff of 30 kDa in a crossflow filtration system. The high-molecular-weight fraction was precipitated by the addition of 4 volumes of 95% ethanol. The mixture was allowed to stand at 4°C overnight and was then centrifuged at 6000 rpm for 20 min to obtain the precipitate. The precipitate was lyophilized to produce HMFO, and the chemical and monosaccharide compositions of HMFO were analyzed in previous study [25]. The molecular weight range of HMFO was estimated to be between 11.8 and 520.4 kDa.

### 2.2. Animals and the Ulcerative Colitis Model

Experiments were performed using 8-week-old female BALB/c mice after a 1-week acclimatization period. The mice were purchased from Koatech Animal Inc. (Pyeongtaek, Korea) and maintained in a temperature- and light-controlled facility with free access to standard rodent chow and water. All experimental protocols were in compliance with the Guiding Principles for the Care and Use of Laboratory Animals of the Ethics Committee of the Korea Food Research Institute as well as internationally accepted principles for laboratory animal use and care as found in the US guidelines (NIH publication number 85-23, revised in 1985). Twenty-five mice were weighed and divided into 5 groups, with 5 mice per group. Mice in group 1 (untreated control) received regular drinking water. Mice in group 2 received 5% (*w*/*v*) DSS (M.W. 36,000–50,000 Da; MP Biomedicals, Solon, OH, USA) in drinking water for 7 days and then received regular water for 3 days. Mice in groups 3 and 4, with DSS-induced colitis, were treated orally with HMFO at 100 and 200 mg/kg, respectively, from day 1 to day 10. Mice in group 5, with DSS-induced colitis, were treated intragastrically with 100 mg/kg 5-ASA as the reference drug. Body weight, food intake, stool consistency, and the presence of gross bleeding were assessed daily, and organ weights and colon lengths were determined after sacrifice.

### 2.3. Disease Activity Index (DAI)

The DAI was calculated by scoring weight loss, diarrhea, and rectal bleeding based on a previously described scoring system [27], as shown in Table 1. Weight loss was defined as the difference between the initial and final weights, and diarrhea was defined as the absence of fecal pellet formation and the presence of continuous fluid fecal material in the colon. Rectal bleeding was assessed based on the presence of visible blood in diarrhea or gross rectal bleeding. DAI values were calculated as (combined score of weight loss, stool constancy, and bleeding)/3. The mice were sacrificed at the end of the experiment, and the colons were separated from the proximal rectum, close to where they passed under the pelvis sternum. The colon length was measured between the ileocecal junction and the proximal rectum.

### 2.4. Assessment of Myeloperoxidase (MPO) Activity

The accumulation of MPO in colon tissues was measured as a marker of neutrophil influx into the colon. Colon tissues were thawed and homogenized in lysis buffer. Subsequently, the homogenates were centrifuged at 1500 ×g for 15 min, and the resulting supernatants were assayed for MPO assay using the colorimetric activity assay kit (Sigma, St. Louis, MO, USA) according to the manufacturer's recommended protocol.

### 2.5. Histopathological Analysis

The entire colon was dissected and flushed with ice-cold phosphate-buffered saline (PBS). Samples of the rectum were obtained and fixed in 10% neutral-buffered formalin (Sigma-Aldrich) for 24 h at room temperature, embedded in paraffin, and sectioned for histological evaluation. The paraffin-embedded tissues were cut into 4 *μ*m sections and stained with hematoxylin and eosin (H&E). The severity of colitis was evaluated in H&E-stained sections by 2 independent observers blinded to the experimental conditions, according to modified criteria [28], and the results are summarized in Table 2. To perform immunohistochemical analysis for TNF-*α* and IL-6 expression in the colon tissue, the 4 *μ*m-thick tissue sections were deparaffinized using xylene and dehydrated in a gradient of alcohol solutions. To exclude endogenous peroxidase activity, the sections were incubated in 0.3% H_2_O_2_ for 15 min and then incubated with primary antibodies (diluted 1 : 200) against proteins of interest for 1 h. The detection system visualized anti-mouse antibodies (K4001; DAKO, Glostrup, Denmark) and was applied according to the manufacturers' instructions. Slides were stained with liquid diaminobenzidine tetrahydrochloride (DAB+), a high-sensitivity substrate-chromogen system (K3468; DAKO). The images on the slides were visualized under an Olympus BX40 light microscope.

### 2.6. Cell Culture and Treatment

RAW 264.7 cells were grown at 37°C and 5% CO_2_ in Dulbecco's modified Eagle's medium (DMEM) containing 10% fetal bovine serum, 100 mg/mL streptomycin, and 100 U/mL penicillin. The cells were seeded in 48-well plates at a density of 2 × 10^5^ cells/well in normal DMEM and pretreated with the indicated concentrations of HMFO for 2 h. Then, the cells were incubated with LPS (1 *μ*g/mL) at 37°C for 22 h.

### 2.7. Measurement of Nitrous Oxide (NO) and Prostaglandin E_2_ (PGE_2_) Production

The inhibitory effects of HMFO on NO and PGE_2_ production were determined using the Griess reagent [29] and enzyme-linked immunosorbent assay (ELISA) kits (R&D Systems, Minneapolis, MN, USA), respectively, according to manufacturer's instructions.

### 2.8. Determination of Tumor Necrosis Factor Alpha (TNF-*α*) and Interleukin-6 (IL-6) Production

Cell culture supernatants and mouse sera were collected, and TNF-*α* and IL-6 levels were measured with ELISA kits (BD Biosciences, San Jose, CA) targeting either protein, according to the manufacturer's protocols.

### 2.9. Western Blot Analysis

Equal amounts of lysates were resolved by sodium dodecyl-polyacrylamide gel electrophoresis and then transferred to a nitrocellulose membrane. The blots were visualized using an enhanced chemiluminescence system (Amersham Biosciences Inc., Piscataway, NJ, USA) followed by exposure to X-ray film (Fuji Photo Film Co. Ltd., Tokyo, Japan), as previously described [25]. Primary antibodies against iNOS, total p38, phosphorylated JNK, phosphorylated ERK1/2, total JNK, total ERK1/2, and *β*-actin were purchased from Santa Cruz Biotechnology (Dallas, TX, USA). Antibodies against phosphorylated I*κ*B-*α*, phosphorylated IKK-*α*/*β*, and phosphorylated p38 were purchased from Cell Signaling Technology Inc. (Beverly, MA, USA).

### 2.10. mRNA Analysis by Semiquantitative RT-PCR

Total cellular RNA was isolated with TRIzol LS (Invitrogen, Carlsbad, CA, USA) according to the manufacturer's instructions. Then, cDNA was synthesized using the cDNA synthesis kit (Thermo Scientific, Sankt Leon-Rot, Germany) according to the manufacturer's protocol. Target-specific RT-PCR primers were designed for iNOS, COX-2, TNF-a, IL-6, and *β*-actin (Table 3).

### 2.11. NF-*κ*B DNA-Binding Assay

RAW 267.4 macrophages were treated with various concentrations of HMFO for 2 h prior to LPS addition. Macrophages were collected 18 h after activation with LPS and washed one time in PBS (pH 7.4). Nuclear extract was isolated using NE-PER nuclear and cytoplasmic extraction reagents according to the manufacturer's instructions (Pierce, Rockford, IL). For detection of NF-*κ*B binding, the nuclear protein was treated according to instructions of by NF-*κ*B (p65) transcription factor assay kit (Abcam, Cambridge, MA, USA). The optical density was determined at 450 nm by an ELISA reader.

## 3. Results and Discussion

### 3.1. Effects of HMFO on the Symptoms of DSS-Induced Colitis

**Figure 1:**
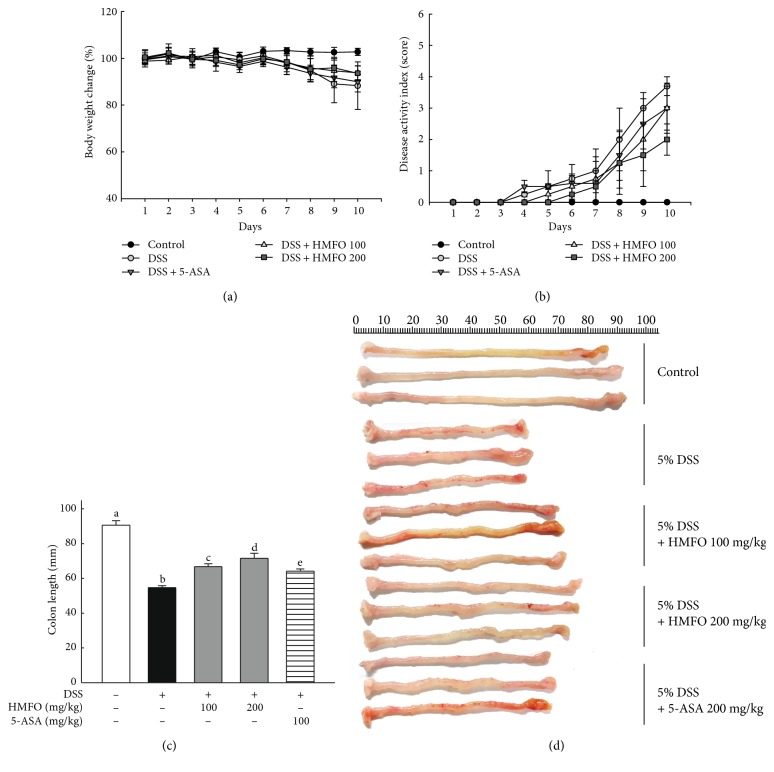
Effects of HMFO on clinical signs and colon lengths in mice with DSS-induced colitis. (a) Changes in body weight and (b) disease activity index scores in the control, DSS, DSS + HMFO 100 (100 mg/kg), DSS + HMFO 200 (200 mg/kg), and DSS+ 5-ASA 100 (100 mg/kg) groups were evaluated daily. (c) The length of each colon was measured and averaged. Values with different letters are significantly different (*p* < 0.05). (d) Colons were removed from DSS-treated mice on day 10, opened longitudinally, washed with PBS, and photographed. 5-ASA, 5-aminosalicylic acid (100 mg/kg); DSS, dextran sulfate sodium; HMFO, polysaccharides of *Cynanchum wilfordii* (100 or 200 mg/kg).

DSS-induced colitis in mice is a well-established preclinical model that exhibits many of the phenotypic features of human ulcerative colitis [30]. On day 10, mice with DSS-induced colitis showed a 12% body weight loss compared to the weight of mice in the control group, while mice treated with 200 mg/kg HMFO showed a 7% loss (Figure 1(a)). Also on day 10, the DAI scores of mice in the DSS-treated group were 4.0 ± 1.00 points higher than those of mice in the control group. Treatment with HMFO attenuated the DSS-mediated increase in DAI scores on day 10 by 3.0 ± 0.8 and 2.0 ± 0.5 points in mice treated with 100 mg/kg or 200 mg/kg HMFO, respectively (Figure 1(b)). Colon length shortening reflects the extent of colon damage that has occurred during acute DSS-induced colitis [31]. The average colon length was approximately 91 mm in the normal control group, whereas it was decreased to 55 mm in the DSS-treated group. Mice treated with high (200 mg/kg) and low (100 mg/kg) doses of HMFO showed significantly longer colon lengths than mice in the DSS-treated group (67 and 73 mm in the groups treated with 100 and 200 mg/kg HMFO, resp., versus 55 mm in the DSS-treated group; Figures 1(c) and 1(d)). These results demonstrated that HMFO treatment reduced the severity of DSS-induced acute colitis.

### 3.2. Effects of HMFO on Histological Changes and MPO Levels in Mice with DSS-Induced Colitis

**Figure 2:**
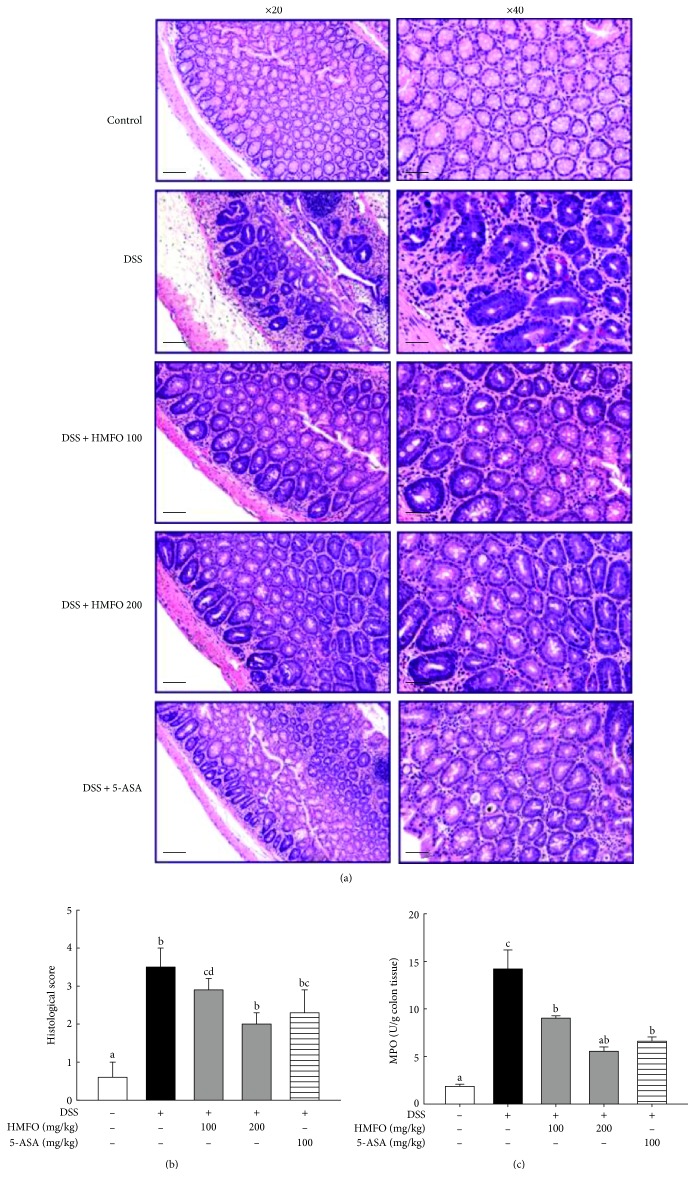
Effects of HMFO on colonic histology and MPO levels in mice with DSS-induced colitis. (a) Histological changes of colonic tissue were determined by hematoxylin and eosin (H&E) staining and observation by light microscopy (×20 and ×40). Left panel: low-power view (scale bar = 100 *μ*m). Right panel: high-power view (scale bar = 50 *μ*m). (b) Histological scores were assigned according to the criteria defined in Section 2.5. (c) MPO was extracted from colon tissue specimens and assayed for colorimetric activity, with the activity determined as the units per gram of colonic tissue. Values with different letters are significantly different (*p* < 0.05). 5-ASA, 5-aminosalicylic acid (100 mg/kg); DSS, dextran sulfate sodium; HMFO, polysaccharides of *Cynanchum wilfordii* (100 or 200 mg/kg); MPO, myeloperoxidase.

Histological analysis was performed to assess the therapeutic effects of HMFO on colonic inflammation. Mucosal thickness is regarded as an indicator of normal mucosal conditions. Treatment with DSS causes epithelial injury and infiltration of inflammatory cells, such as mast cells [32]. Colonic inflammation and mucosal injury were assessed by pathological examination of the colon after H&E staining, and representative results are shown in Figure 2(a). Representative colon tissue sections from mice in the control group showed intact surface epithelium, cryptal glands, stroma, and submucosa. However, colon sections from DSS-treated mice showed branched crypts, cryptitis, decreased numbers of crypts and goblet cells, inflammatory cell infiltration, and extensive submucosal edema. In contrast, HMFO treatment improved the signs of histological damage, such as abnormal crypts, crypt loss, and inflammatory cell infiltration (Figure 2(a)). Mice treated with DSS alone had significantly elevated histological scores, compared with those of healthy control mice. However, the histological score decreased to approximately 57% of that in the DSS-treated group when treated with the highest HMFO concentration (200 mg/kg) (Figure 2(b)). These results suggested that HMFO treatment protected against acute DSS-induced colitis. To evaluate the protective effects of HMFO against leukocyte infiltration in colonic tissue, we measured the levels of MPO, a marker of leukocyte infiltration in the colon tissue. MPO is an enzyme that is produced mainly by polymorphonuclear leukocytes and is specific to granulocyte lysosomes. Therefore, MPO is directly correlated with the number of neutrophils [33]. As shown in Figure 2(c), DSS-treated mice showed a significant increase in MPO activity compared to that in control mice, and this increase was drastically decreased in the HMFO-treated group. Therefore, the increase in MPO activity in tissues induced by DSS correlated with the development of colonic inflammation, and HMFO administration markedly suppressed MPO accumulation in colonic tissues.

### 3.3. Effects of HMFO on Serum Levels and Immunohistochemical Staining for Proinflammatory Cytokines

**Figure 3:**
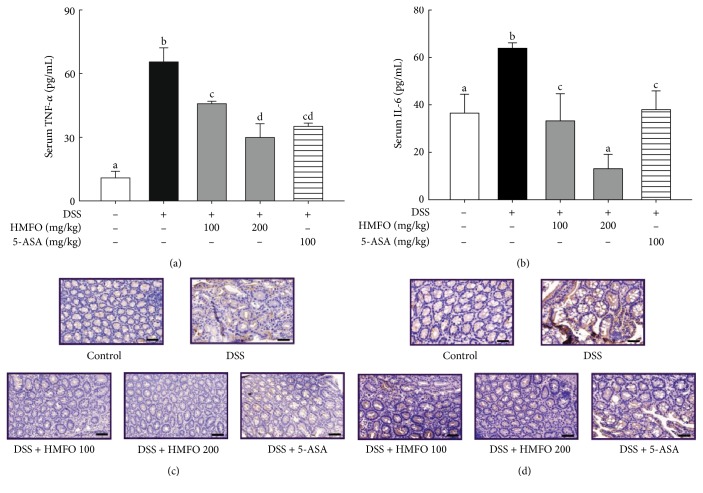
Effects of HMFO on serum levels and immunohistochemical staining for proinflammatory cytokines. (a) TNF-*α* production in mouse sera on day 10. (b) IL-6 production in mouse sera on day 10. Immunohistochemical staining of TNF-*α* (c) and IL-6 (d) in colonic tissue (×40, scale bar = 50 *μ*m). Brown staining indicates TNF-*α* (c) and IL-6 (d) positivity, respectively. Values with different letters are significantly different (*p* < 0.05). 5-ASA, 5-aminosalicylic acid (100 mg/kg); DSS, dextran sulfate sodium; HMFO, polysaccharides of *Cynanchum wilfordii* (100 or 200 mg/kg).

A key characteristic of DSS-induced colitis is the increased secretion of proinflammatory cytokines into the serum [34, 35]; thus, we measured serum cytokine levels in this study. Serum IL-6 levels were significantly higher in the DSS-treated group than in the control group. However, IL-6 levels in mice treated with HMFO (100 and 200 mg/kg) were lower than those in the DSS-treated group (Figure 3(a)). Serum TNF-*α* levels were also increased in DSS-treated mice compared with the normal control group, while HMFO-treated mice had lower serum TNF-*α* concentrations than DSS-treated mice (Figure 3(b)). Moreover, immunohistochemistry analysis indicated that HMFO at 200 mg/kg markedly reversed the increase in IL-6- and TNF-*α*-positive cells (brown stained) in the colonic mucosa of DSS-induced mice (Figures 3(c) and 3(d)). High levels of proinflammatory cytokine production are a major characteristic of DSS-induced colitis [34, 35]. In DSS-induced acute colitis, enormous infiltrates appeared in inflammatory lesions, mainly consisting of T and B lymphocytes, macrophages, and neutrophils, which produce various proinflammatory cytokines, including TNF-*α*, IFN-*γ*, IL-6, IL-8, IL-12, and IL-17 [34, 36, 37]. In the colon of patients with IBD, markedly increased numbers of mast cells and macrophages were observed, and these cells might contribute to abnormal production of inflammatory cytokines and mediators, such as NO and TNF-*α* [38].

### 3.4. Effects of HMFO on iNOS and COX-2 Expression in the Colon of DSS-Treated Mice

**Figure 4:**
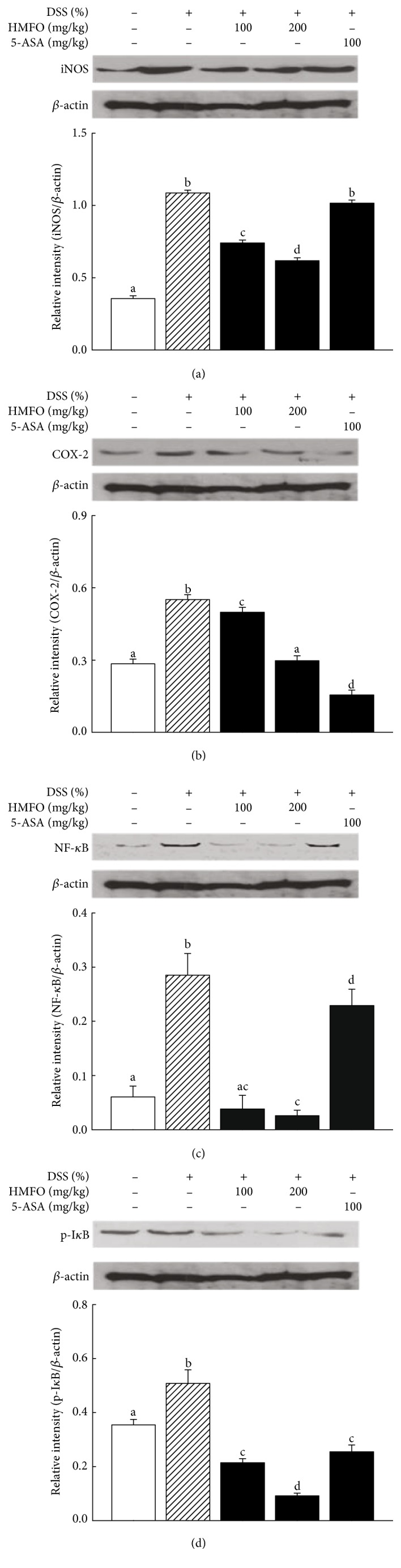
Effects of HMFO on iNOS, COX-2, NF-*κ*B, and p-I*κ*B protein expression in mice with DSS-induced colitis. Western blot analysis was used to determine (a) iNOS, (b) COX-2, (c) NF-*κ*B, and (d) p-I*κ*B levels in colonic tissues. The relative ratios of iNOS, COX-2, and the phosphorylated NF-*κ*B p65 subunit to *β*-actin were calculated using an image analyzer. Values with different letters are significantly different (*p* < 0.05). 5-ASA, 5-aminosalicylic acid (100 mg/kg); DSS, dextran sulfate sodium; HMFO, polysaccharides of *Cynanchum wilfordii* (100 or 200 mg/kg).

The effects of HMFO on the expression of inflammatory proteins such as COX-2 and iNOS in cytosolic extracts from the colon of colitis mice were detected by Western blot analysis. As shown in Figures 4(a) and 4(b), iNOS and COX-2 expression levels significantly increased in the colon tissues from DSS-treated mice compared to those in normal mice. However, HMFO administration remarkably reduced iNOS protein upregulation and significantly decreased DSS-induced COX-2 expression, down to normal levels with different doses of HMFO (Figures 4(a) and 4(b)). The COX-2 and iNOS enzymes represent important molecular targets in the treatment and prevention of IBD, and their expression and activity are associated with disease severity, suggesting their potential as anti-inflammation drug targets. During active inflammation, bacteria can enter the lamina propria due to cell destruction and increased permeability. Inflammatory cytokines and bacterial antigens induce and drive the transcription of both COX-2 and iNOS, and these proinflammatory enzymes are also upregulated in murine experimental colitis and active human IBD [39, 40]. Our data clearly demonstrated that colonic damage was associated with elevated expression of both COX-2 and iNOS proteins, and HMFO administration potently downregulated their expression levels. In a similar study, pomegranate polyphenols and resveratrol decreased COX-2 and iNOS overexpression in murine experimental colitis [41, 42].

### 3.5. Effect of HMFO on NF-*κ*B p65 Subunit Phosphorylation in Mice with DSS-Induced Colitis

NF-*κ*B is a major transcription factor that, by regulating the expression of multiple inflammatory and immune genes, plays a key role in host defense and chronic inflammatory diseases [43]. NF-*κ*B was activated in the mucosal cells of IBD patients [44] and in experimental colitis models [41] and, thus, may be an ideal target for UC therapy. Therefore, the effect of HMFO on NF-*κ*B activation in mice with DSS-induce colitis was investigated. DSS administration significantly elevated the phosphorylated NF-*κ*B p65 levels in the colon tissue compared to those in the control group. However, HMFO treatment drastically attenuated NF-*κ*B phosphorylation, which was comparable in HMFO-treated and control mice (Figure 4(c)). These results suggested that HMFO can inhibit the activation of transcription factors in mice with DSS-induced colitis and may be an effective and promising treatment for UC.

### 3.6. HMFO Inhibited LPS-Induced NO Production by Suppressing iNOS Expression in RAW 264.7 Macrophages

**Figure 5:**
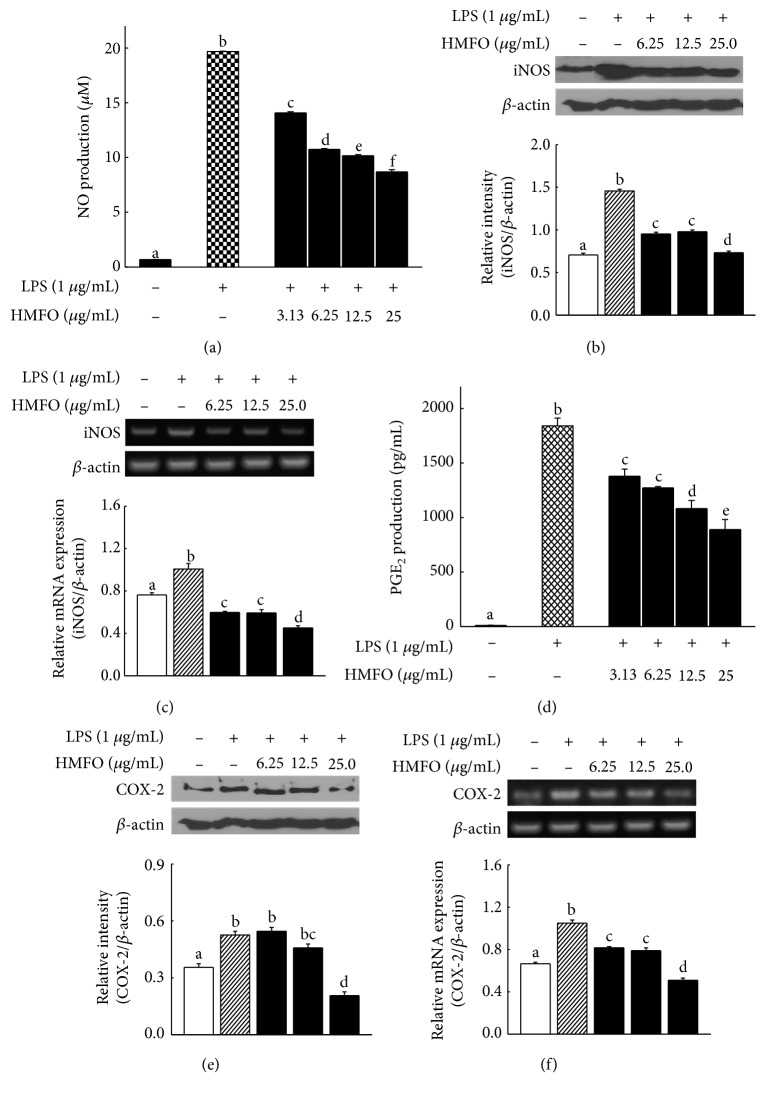
HMFO inhibited LPS-induced inflammatory mediators by suppressing iNOS and COX-2 activation in RAW 264.7 macrophages. RAW 264.7 macrophages were pretreated with different HMFO concentrations for 2 h and then stimulated with LPS (1 *μ*g/mL) for 22 h. (a) Supernatant NO concentrations were calculated by comparing the measured values to those of standard concentrations of sodium nitrite dissolved in DMEM. (b, e) Cell lysates were obtained, and iNOS and COX-2 protein levels were analyzed by Western blotting. (c, f) Total RNA was isolated, and iNOS and COX-2 mRNA levels were measured by RT-PCR. (d) PGE_2_ was quantified using an ELISA kit according to the manufacturer's protocol. Values with different letters are significantly different (*p* < 0.05).

The effects of HMFO on LPS-induced NO production in RAW 264.7 macrophages were investigated by measuring NO release into the culture supernatant, using the Griess reagent. As shown in Figure 5(a), HMFO significantly decreased LPS-induced NO production in a dose-dependent manner, with >58% inhibition at 25 *μ*g/mL HMFO, a nontoxic concentration. NO is synthesized by iNOS and is a well-known proinflammatory mediator involved in various physiological and pathological processes. Suppression of NO production has been suggested as a new pharmacological strategy for treating inflammation-related diseases [45]. As shown in Figures 5(b) and 5(c), iNOS expression at both the mRNA and protein levels significantly increased following LPS stimulation. However, iNOS mRNA and protein levels in LPS-stimulated RAW 264.7 macrophages were significantly reduced by treatment with HMFO. These results indicated that HMFO suppressed NO production by suppressing iNOS gene expression in LPS-induced RAW 264.7 macrophages. The iNOS gene is the primary regulator of NO production in macrophages, and iNOS inhibitors have been found to attenuate osteoarthritis [46], periodontitis [47], septic shock [48], and other chronic inflammatory diseases.

### 3.7. HMFO Inhibited LPS-Induced PGE_2_ Production by Suppressing COX-2 Expression in RAW 264.7 Macrophages

We examined whether PGE_2_ production was suppressed by HMFO in LPS-stimulated RAW 264.7 cells. LPS (1 *μ*g/mL) significantly increased PGE_2_ production compared to the basal production level in the absence of LPS. Treatment with HMFO significantly inhibited LPS-induced PGE_2_ production in a concentration-dependent manner (Figure 5(d)). COX-2 mRNA expression and protein levels increased markedly in response to LPS (1 *μ*g/mL), and treatment with HMFO inhibited LPS-activated COX-2 gene and protein expression in a dose-dependent manner (Figures 5(e) and 5(f)). PGE_2_, which is generated by COX-2, plays important roles in the inflammatory response, and LPS treatment resulted in elevated PGE_2_ levels at inflammation sites [49]. COX-2 can also be activated by high concentrations of NO, which intensifies the inflammatory responses in many chronic inflammatory disorders [50].

### 3.8. HMFO Inhibited LPS-Stimulated TNF-*α* and IL-6 Production in RAW 264.7 Macrophages

**Figure 6:**
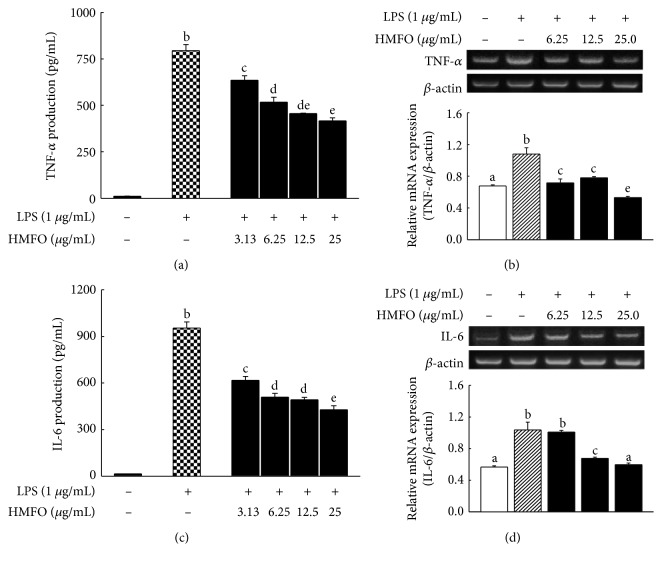
Effects of HMFO on LPS-induced proinflammatory cytokine production and mRNA expression in RAW 264.7 macrophages. RAW 264.7 macrophages were pretreated with different HMFO concentrations for 2 h and then stimulated with LPS (1 *μ*g/mL) for 22 h. (a) TNF-*α* and (c) IL-6 were quantified with an ELISA kit, according to the manufacturer's protocol. The data are presented as the mean ± SD. Total RNA was isolated, and (b) TNF-*α* and (d) IL-6 mRNA levels were measured by RT-PCR. Values with different letters are significantly different (*p* < 0.05).

To examine the ability of HMFO to inhibit the expression of major proinflammatory cytokines, we measured TNF-*α* and IL-6 production in untreated and HMFO-pretreated, LPS-stimulated RAW 264.7 macrophages. Treatment with HMFO inhibited LPS-induced TNF-*α* (Figure 6(a)) and IL-6 production (Figure 6(c)) in a dose-dependent manner. We next investigated the effects of HMFO on LPS-induced TNF-*α* and IL-6 gene expression in RAW 264.7 macrophages. The mRNA levels of TNF-*α* and IL-6 were upregulated by LPS stimulation, but treatment with HMFO suppressed TNF-*α* (Figure 6(b)) and IL-6 (Figure 6(d)) mRNA expression, implying that HMFO could block transcriptional activation induced by inflammation-regulating transcription factors, such as NF-*κ*B and AP-1 [51].

### 3.9. Inhibitory Effects of HMFO on LPS-Induced NF-*κ*B Activation in RAW 264.7 Macrophages

**Figure 7:**
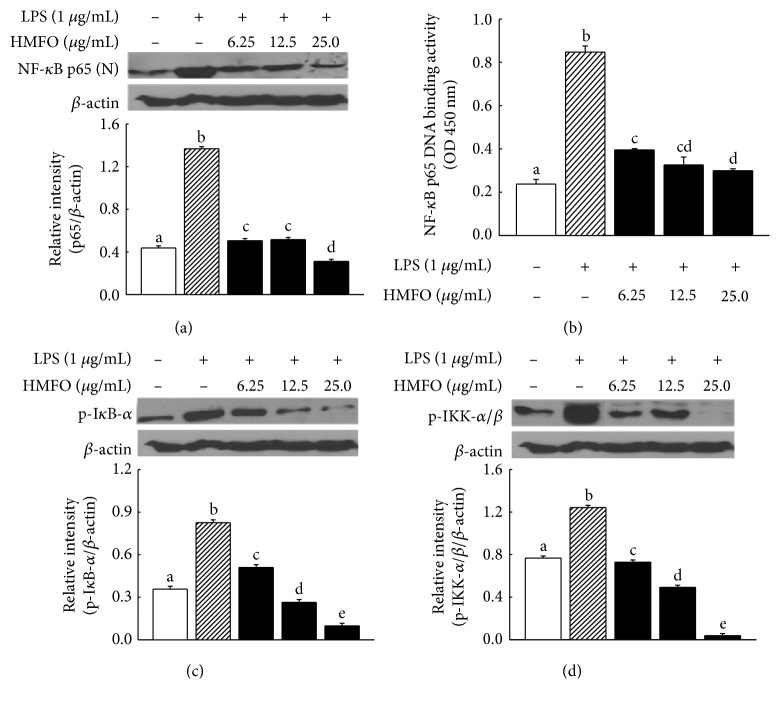
Effects of HMFO on the NF-*κ*B signaling pathways in LPS-induced RAW 264.7 macrophages. RAW 264.7 macrophages were pretreated with different concentrations of HMFO for 2 h and then stimulated with LPS for 22 h. Phosphorylation of (a) p65, (b) I*κ*B-*α*, and (c) IKK *α*/*β* was analyzed by Western blotting. The NF-*κ*B DNA binding activies (b) were measured by NF-*κ*B (p65) transcription factor assay kit as described in Materials and Methods. Values with different letters are significantly different (*p* < 0.05).

To examine the inhibitory effects of HMFO on NF-*κ*B activation, we measured nuclear NF-*κ*B and cytoplasmic IKK *α*/*β* and I*κ*B *α*/*β* levels by Western blotting. As shown in Figure 7(a), stimulation of RAW 264.7 macrophages with LPS increased nuclear translocation of NF-*κ*B; however, this effect was inhibited by HMFO treatment. Our findings indicate that HMFO decreased NF-*κ*B activation by blocking the nuclear translocation of p65 via IKK-*α*/*β*-dependent I*κ*B phosphorylation (Figures 7(c) and 7(d)). NF-*κ*B is a ubiquitous transcription factor that is constitutively expressed in many cell types, including cells of the monocyte/macrophage lineage [52]. Nuclear translocation of NF-*κ*B is involved in initiating the transcription of several genes, including TNF-*α* and iNOS [53].

### 3.10. Inhibitory Effects of HMFO on LPS-Induced MAPK Activation in RAW 264.7 Macrophages

**Figure 8:**
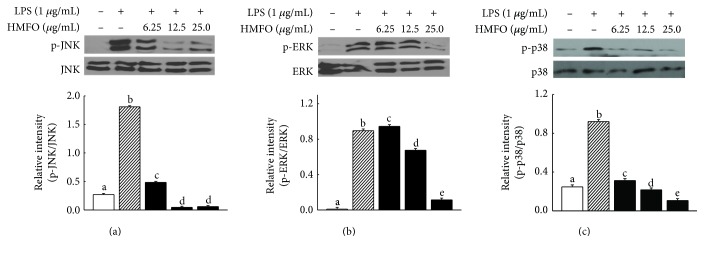
Effects of HMFO on MAPK signaling pathways in LPS-induced RAW 264.7 macrophages. RAW 264.7 macrophages were pretreated with different concentrations of HMFO for 2 h and then stimulated with LPS for 22 h. MAPKs and phosphorylated MAPKs were detected by Western blot analysis using antibodies against the corresponding MAPKs and phosphorylated (a) JNK, (b) ERK, and (c) p38 (activated forms), as described in Section 2.9. Values with different letters are significantly different (*p* < 0.05).

MAPKs play critical roles in regulating cell growth and differentiation, cellular responses to cytokines [54], and modulating NF-*κ*B activity [55]. To investigate whether HFMO-dependent inhibition of NF-*κ*B activation and NO production occurred through the MAPK pathway, we examined the effects of HMFO on the LPS-induced phosphorylation of JNK, p38, and ERK by Western blot analysis. As shown in Figures 8(a) and 8(c), pretreating cells with HMFO inhibited LPS-induced phosphorylation of JNK, ERK, and p38 in a concentration-dependent manner. These results suggest that HMFO may inhibit NF-*κ*B activation and NO production in RAW 264.7 macrophages by suppressing MAPK phosphorylation.

## 4. Conclusion

In summary, our results suggest that HMFO inhibited the expression of several cytokines and enzymes involved in inflammation, such as PGE_2_, NO, TNF-*α*, IL-6, iNOS, and COX-2 by attenuating NF-*κ*B and MAPKs in RAW 264.7 macrophages. Furthermore, HMFO ameliorated the pathological characteristics of colitis, such as shortened colon length, and significantly reduced the serum levels of proinflammatory cytokines. Histological analysis indicated that HMFO improved the signs of histological damage such as abnormal crypts, crypt loss, and inflammatory cell infiltration induced by DSS. In addition, HMFO inhibited iNOS and COX-2 protein expression and the levels of phosphorylated NF-*κ*B p65 in the colon tissue of mice with DSS-induced colitis. These results indicated that HMFO attenuated inflammation both in vitro and in vivo primarily by inhibiting NF-*κ*B activation. Thus, our findings indicate that HMFO is an effective and promising remedy for IBDs, such as colitis.

## Figures and Tables

**Table 1:** Criteria for scoring the DAI^(1)^.

Score	Weight loss (%)	Stool consistency^(2)^	Bloodstain or gross bleeding
0	None	Normal	Negative
1	1–5	Loose stool	Negative
2	5–10	Loose stool	Positive
3	10–15	Diarrhea	Positive
4	>15	Diarrhea	Gross bleeding

^(1)^Disease activity index = (combined score of weight loss, stool constancy, and bleeding)/3. ^(2)^Normal stool = well-formed pellets; loose stool = pasty stool that did not stick to the anus; and diarrhea = liquid stool that stuck to the anus.

**Table 2:** Criteria for assessment of microscopic rectal damage.

Score	Observation
0	Normal colonic mucosa
1	Loss of one-third of the crypts
2	Loss of two-thirds of the crypts
3	Lamina propria covered with a single layer of epithelial cells, with mild inflammatory cell infiltration
4	Erosions and marked inflammatory cell infiltration

Mucosal damage was scored 0–4 based on the loss of crypts (mucosa) and infiltration of inflammatory cells (maximum score = 4).

**Table 3:** PCR primers used in this study.

Gene		Sequence (5′–3′)
iNOS	Sense	ACCCAAGGTCTACGTTCAGG
	Antisense	CGCACATCTCCGCAAATGTA
COX-2	Sense	CCTGAGCATCTACGGTTTGC
	Antisense	ACTGCTCATCACCCCATTCA
TNF-*α*	Sense	AGGGGAAATGAGAGACGCAA
	Antisense	TTCCCCATCTCTTGCCACAT
IL-6	Sense	CCGGAGAGGGACTTCACAG
	Antisense	GGAAATTGGGGTAGGAAGGA
*β*-Actin	Sense	ACTCTTCCAGCCTTCCCTCC
	Antisense	CGTACAGGTCTTTGCGGATG

## Abbreviations

DAI: Disease activity index
DSS: Dextran sulfate sodium
LPS: Lipopolysaccharide
NF-*κ*B: Nuclear factor-*κ*B
IBD: Inflammatory bowel disease
UC: Ulcerative colitis
TNF-*α:*: Tumor necrosis factor-*α*
IL: Interleukin
iNOS: Inducible nitric oxide synthase
COX-2: Cyclooxygenase-2
NO: Nitric oxide
MPO: Myeloperoxidase
PGE_2_: Prostaglandin E_2_.

## References

[1] Hui D. S., Zumla A. (2014). Advancing priority research on the Middle East respiratory syndrome coronavirus. *The Journal of Infectious Diseases*.

[2] Loomis D., Grosse Y., Lauby-Secretan B. (2013). The carcinogenicity of outdoor air pollution. *The Lancet Oncology*.

[3] Head K. A., Jurenka J. S. (2003). Inflammatory bowel disease part 1: ulcerative colitis—pathophysiology and conventional and alternative treatment options. *Alternative Medicine Review*.

[4] Munkholm P., Langholz E., Hollander D. (1994). Intestinal permeability in patients with Crohn’s disease and ulcerative colitis and their first degree relatives. *Gut*.

[5] Truelove S. C., Horler A. R., Richards W. (1955). Serial biopsy in ulcerative colitis. *British Medical Journal*.

[6] Lix L. M., Graff L. A., Walker J. R. (2008). Longitudinal study of quality of life and psychological functioning for active, fluctuating, and inactive disease patterns in inflammatory bowel disease. *Inflammatory Bowel Diseases*.

[7] Opheim R., Bernklev T., Fagermoen M. S., Cvancarova M., Moum B. (2012). Use of complementary and alternative medicine in patients with inflammatory bowel disease: results of a cross-sectional study in Norway. *Scandinavian Journal of Gastroenterology*.

[8] Oxelmark L., Lindberg A., Löfberg R. (2016). Use of complementary and alternative medicine in Swedish patients with inflammatory bowel disease: a controlled study. *European Journal of Gastroenterology & Hepatology*.

[9] Rawsthorne P., Clara I., Graff L. A. (2012). The Manitoba inflammatory bowel disease cohort study: a prospective longitudinal evaluation of the use of complementary and alternative medicine services and products. *Gut*.

[10] Weizman A. V., Ahn E., Thanabalan R. (2012). Characterisation of complementary and alternative medicine use and its impact on medication adherence in inflammatory bowel disease. *Alimentary Pharmacology & Therapeutics*.

[11] Tsiang Y., Li P. T. (1977). Asclepiadaceae. *Delectis Florae Reipublicae Popularis Sinicae Agendae Academiae Sinicae*.

[12] Chang A., Kwak B. Y., Yi K., Kim J. S. (2012). The effect of herbal extract (EstroG-100) on pre-, peri- and post-menopausal women: a randomized double-blind, placebo-controlled study. *Phytotherapy Rresearch*.

[13] Zenk M. H., el-Shagi H., Schulte U. (1975). Anthraquinone production by cell suspension cultures of Morinda citrifolia. *Planta Medica*.

[14] Zhang X., Shan L., Huang H. (2009). Rapid identification of acetophenones in two Cynanchum species using liquid chromatography-electrospray ionization tandem mass spectrometry. *Journal of Pharmaceutical and Biomedical Analysis*.

[15] Korea Food and Drug Administration (KFDA) http://www.mfds.go.kr/herbmed/index.do?nMenuCode=7&code=KHP-N126&includeUrl=/herbmed/view.jsp.

[16] Choi D. H., Lee Y. J., Oh H. C. (2012). Improved endothelial dysfunction by *Cynanchum wilfordii* in apolipoprotein E(−/−) mice fed a high fat/cholesterol diet. *Journal of Medicinal Food*.

[17] Hwang B. Y., Kim S. E., Kim Y. H. (1999). Pregnane glycoside multidrug-resistance modulators from *Cynanchum wilfordii*. *Journal of Natural Products*.

[18] Kim H. S. (2004). Effects of *Cynanchum wilfordii* extract on serum lipid components and enzyme activities in hyperlipidemic and streptozotocin-induced diabetic rats. *Korean Journal of Human Ecology*.

[19] Niu J. Z., Ye B. K., Wang D. F. (1998). Observation of the protection effect of Baishouwu to the liver of the high serum cholesterol mouse. *Ji Sheng Chong Yu Yi Xue kun Chong Xue Bao*.

[20] Shan L., Liu R. H., Shen Y. H. (2006). Gastroprotective effect of a traditional Chinese herbal drug “baishouwu” on experimental gastric lesions in rats. *Journal of Ethnopharmacology*.

[21] Shan L., Zhang W. D., Zhang C., Liu R. H., Su J., Zhou Y. (2005). Antitumor activity of crude extract and fractions from root tuber of *Cynanchum auriculatum* Royle ex Wight. *Phytotherapy Research*.

[22] Yoon M. Y., Choi N. H., Min B. S. (2011). Potent in vivo antifungal activity against powdery mildews of pregnane glycosides from the roots of *Cynanchum wilfordii*. *Journal of Agricultural and Food Chemistry*.

[23] Srivastava R., Kulshreshtha D. K. (1989). Bioactive polysaccharides from plants. *Phytochemistry*.

[24] You L., Gao Q., Feng M. (2013). Structural characterisation of polysaccharides from *Tricholoma matsutake* and their antioxidant and antitumour activities. *Food Chemistry*.

[25] Jang M., Lim T. G., Ahn S. (2016). Immune-enhancing effects of a high molecular weight fraction of *Cynanchum wilfordii* Hemsley in macrophages and immunosuppressed mice. *Nutrients*.

[26] Dore C. M., das C Faustino Alves M. G., Will L. S. (2013). A sulfated polysaccharide, fucans, isolated from brown algae Sargassum vulgare with anticoagulant, antithrombotic, antioxidant and anti-inflammatory effects. *Carbohydrate Polymers*.

[27] Murthy S. N., Cooper H. S., Shim H., Shah R. S., Ibrahim S. A., Sedergran D. J. (1993). Treatment of dextran sulfate sodium induced murine colitis by intracolonic cyclosporine. *Digestive Diseases and Sciences*.

[28] Hamamoto N., Maemura K., Hirata I., Murano M., Sasaki S., Katsu K. (1999). Inhibition of dextran sulphate sodium (DSS)-induced colitis in mice by intracolonically administered antibodies against adhesion molecules (endothelial leucocyte adhesion molecule-1 (ELAM-1) or intercellular adhesion molecule-1 (ICAM-1)). *Clinical and Experimental Immunology*.

[29] Sun J., Zhang X., Broderick M., Fein H. (2003). Measurement of nitric oxide production in biological systems by using Griess reaction assay. *Sensors*.

[30] Okayasu I., Hatakeyama S., Yamada M., Ohkusa T., Inagaki Y., Nakaya R. (1990). A novel method in the induction of reliable experimental acute and chronic ulcerative colitis in mice. *Gastroenterology*.

[31] Hendrickson B. A., Gokhale R., Cho J. H. (2002). Clinical aspects and pathophysiology of inflammatory bowel disease. *Clinical Microbiology Reviews*.

[32] Kim D. S., Ko J. H., Jeon Y. D. (2013). *Ixeris dentate* NAKAI reduces clinical score and HIF-1 expression in experimental colitis in mice. *Evidence-based Complementary and Alternative Medicine*.

[33] Kurutas E. B., Cetinkaya A., Bulbuloglu E., Kantarceken B. (2005). Effects of antioxidant therapy on leukocyte myeloperoxidase and Cu/Zn-superoxide dismutase and plasma malondialdehyde levels in experimental colitis. *Mediators of Inflammation*.

[34] Egger B., Bajaj-Elliott M., MacDonald T. T., Inglin R., Eysselein V. E., Büchler M. W. (2000). Characterisation of acute murine dextran sodium sulphate colitis: cytokine profile and dose dependency. *Digestion*.

[35] Garside P. (1999). Cytokines in experimental colitis. *Clinical and Experimental Immunology*.

[36] Ito R., Shin-Ya M., Kishida T. (2006). Interferon-gamma is causatively involved in experimental inflammatory bowel disease in mice. *Clinical and Experimental Immunology*.

[37] Taghipour N., Molaei M., Mosaffa N. (2016). An experimental model of colitis induced by dextran sulfate sodium from acute progresses to chronicity in C57BL/6: correlation between conditions of mice and the environment. *Gastroenterology and Hepatology from bed to Bench*.

[38] Crowe S. E., Perdue M. H. (1992). Functional abnormalities in the intestine associated with mucosal mast cell activation. *Regional Immunology*.

[39] Cirillo C., Sarnelli G., Esposito G. (2009). Increased mucosal nitric oxide production in ulcerative colitis is mediated in part by the enteroglial-derived S100B protein. *Neurogastroenterology and Motility*.

[40] Talero E., Sánchez-Fidalgo S., de la Lastra C. A., Illanes M., Calvo J. R., Motilva V. (2008). Acute and chronic responses associated with adrenomedullin administration in experimental colitis. *Peptides*.

[41] Rosillo M. A., Sánchez-Hidalgo M., Cárdeno A. (2012). Dietary supplementation of an ellagic acid-enriched pomegranate extract attenuates chronic colonic inflammation in rats. *Pharmacological Research*.

[42] Sánchez-Fidalgo S., Cárdeno A., Villegas I., Talero E., de la Lastra C. A. (2010). Dietary supplementation of resveratrol attenuates chronic colonic inflammation in mice. *European Journal of Pharmacology*.

[43] Barnes P. J. (1997). Nuclear factor-kappa B. *The International Journal of Biochemistry & Cell Biology*.

[44] Schreiber S., Nikolaus S., Hampe J. (1998). Activation of nuclear factor kappa B inflammatory bowel disease. *Gut*.

[45] Tinker A. C., Wallac A. V. (2006). Selective inhibitors of inducible nitric oxide synthase: potential agents for the treatment of inflammatory diseases?. *Current Topics in Medicinal Chemistry*.

[46] Pelletier J. P., Jovanovic D., Fernandes J. C. (1998). Reduced progression of experimental osteoarthritis in vivo by selective inhibition of inducible nitric oxide synthase. *Arthritis and Rheumatism*.

[47] Lohinai Z., Benedek P., Fehér E. (1998). Protective effects of mercaptoethylguanidine, a selective inhibitor of inducible nitric oxide synthase, in ligature-induced periodontitis in the rat. *British Journal of Pharmacology*.

[48] Levy B., Valtier M., De Chillou C., Bollaert P. E., Cane D., Mallie J. P. (1999). Beneficial effects of L-canavanine, a selective inhibitor of inducible nitric oxide synthase, on lactate metabolism and muscle high energy phosphates during endotoxic shock in rats. *Shock*.

[49] Yoon W. J., Ham Y. M., Yoo B. S., Moon J. Y., Koh J., Hyun C. G. (2009). *Oenothera laciniata* inhibits lipopolysaccharide induced production of nitric oxide, prostaglandin E_2_, and proinflammatory cytokines in RAW264.7 macrophages. *Journal of Bioscience and Bioengineering*.

[50] Ibrahim Abdelwahab S., Syaed Koko W., Mohamed Elhassan Taha M. (2012). *In vitro* and *in vivo* anti-inflammatory activities of columbin through the inhibition of cycloxygenase-2 and nitric oxide but not the suppression of NF-κB translocation. *European Journal of Pharmacology*.

[51] Zhang G., Ghosh S. (2000). Molecular mechanisms of NF-kappaB activation induced by bacterial lipopolysaccharide through Toll-like receptors. *Journal of Endotoxin Research*.

[52] Frankenberger M., Pforte A., Sternsdorf T., Passlick B., Baeuerle P. A., Ziegler-Heitbrock H. W. (1994). Constitutive nuclear NF-kappa B in cells of the monocyte lineage. *The Biochemical Journal*.

[53] Müller J. M., Ziegler-Heitbrock H. W., Baeuerle P. A. (1993). Nuclear factor kappa B, a mediator of lipopolysaccharide effects. *Immunobiology*.

[54] Vanden Berghe W., Plaisance S., Boone E. (1998). p38 and extracellular signal-regulated kinase mitogen-activated protein kinase pathways are required for nuclear factor-kappaB p65 transactivation mediated by tumor necrosis factor. *The Journal of Biological Chemistry*.

[55] Surh Y. J., Chun K. S., Cha H. H. (2001). Molecular mechanisms underlying chemopreventive activities of anti-inflammatory phytochemicals: down-regulation of COX-2 and iNOS through suppression of NF-kappa B activation. *Mutation Research*.

